# Primary Anal Squamous Cell Carcinoma With Metastasis to the Brain Presenting As Stroke-Like Symptoms in a 27-Year-Old Patient

**DOI:** 10.7759/cureus.105314

**Published:** 2026-03-16

**Authors:** Mam Jarra Gai, Chukwunonso C Ndulue, Kristin N Slater, Athina Amanor, Wouhabe Bancheno

**Affiliations:** 1 Ophthalmology, Howard University Hospital, Washington, D.C., USA; 2 Internal Medicine, Howard University Hospital, Washington, D.C., USA; 3 Internal Medicine, Howard University College of Medicine, Washington, D.C., USA

**Keywords:** brain metastases, brain metastases in anal scc, hpv 16, human papillomavirus (hpv), invasive moderately differentiated squamous cell carcinoma, metastatic anal squamous cell carcinoma, squamous cell carcinoma (scc), stroke-like symptoms, vasogenic brain edema, whole brain radiation

## Abstract

Metastatic squamous cell carcinoma (SCC) with spread to the brain is a rare entity in primary anal SCC. These findings are especially unusual in a young, HIV-negative patient. We present a case of a 27-year-old woman with metastatic SCC of the anus, previously treated with pelvic radiation and chemotherapy, who presented with acute right-sided weakness mimicking stroke-like symptoms. Neuroimaging revealed multifocal bilateral enhancing lesions consistent with metastatic brain disease. Symptoms resolved following treatment of the vasogenic edema secondary to brain metastasis. This case highlights the unique finding of primary anal SCC with metastasis to the brain in a young, HIV-negative, human papillomavirus type 16 (HPV 16)-positive patient. It further underscores the importance of preventative care with HPV vaccinations to prevent HPV 16 and 18, as well as brain imaging to rule out intracranial metastases in patients with malignancies presenting with new neurological deficits.

## Introduction

Brain metastases are a frequent complication of systemic malignancies, encountered in approximately 10% to 20% of adult cancer patients during the course of disease [1-2]. However, the incidence of brain metastasis varies widely by primary tumor type: lung, breast, melanoma, and renal cell carcinoma collectively account for the majority of cases [1-2]. In contrast, gastrointestinal (GI) tract malignancies are much less commonly associated with intracranial spread [3]. Primary anal cancer is rare, representing merely 2% to 4% of GI cancers [4-8]. Distal metastasis of anal cancer is uncommon, and the presence of brain metastasis is even more rare [4-8]. Squamous cell carcinoma (SCC) makes up the majority of anal cancers, which in some cases is thought to be related to human papilloma virus (HPV) subtypes 16 and 18 [4-5,7]. Brain metastasis of anal SCC is rarely reported in the literature [4-8]. The recent case report by Popa et al. in 2025 noted 10 cases in their review, including their patient, the youngest of whom was 44 years old and the oldest of whom was 69 [4]. To our knowledge, this is the first reported case of an HIV-negative patient in their 20s.

## Case presentation

A 27-year-old female patient with a medical history of beta thalassemia minor, pre-diabetes, hyperlipidemia, a current history of metastatic SCC of the anus (diagnosed at age 26), and cervical Papanicolaou (Pap) testing showing HPV 16 positivity and HPV 18 negativity presented to the emergency department. Her previous extended oncologic history indicated that at 26 years of age, she had initially presented with severe and constant anal pain and spotting with bowel movements. The blood was noticed when wiping instead of in the stool, and she was found to have an anal mass. A biopsy was attained, and immunohistochemical stains were performed, which revealed malignant cells positive for p63, p16, and CK5/6 and negative for BerEP4. It was noted that the P53 stain showed a wild-type pattern. The biopsy supported the diagnosis of HPV-associated invasive moderately differentiated SCC. Additionally, around that time, she had a painful vulvar lesion, which was concerning for malignancy. Four biopsies were taken (left superior vulva, left vulva at 9 o’clock, right vulva at 9 o’clock, and perianal area), all of which showed invasive, moderately differentiated SCC. Shortly thereafter, she was found to have metastasis to the liver, which was confirmed on biopsy. She was determined to have invasive anal SCC with advancements to the vulva and metastases to the liver; she underwent a diversion colostomy, port placement, and two cycles of carboplatin and paclitaxel chemotherapy, which were discontinued due to recurrent port infections. Then she received pelvic radiation (chemoradiotherapy 54 Gy total over the span of two months). Following pelvic radiation therapy, she completed three more months of carboplatin and paclitaxel chemotherapy, then discontinued it for an unknown reason and started nivolumab for two months. Following her discontinuation of nivolumab, she proceeded with the FOLFOX (folinic acid, fluorouracil, and oxaliplatin) chemotherapy regimen, which she continued up to her hospitalization. 

She presented to our hospital’s emergency department with sudden-onset right-sided weakness and heaviness while at work. She denied loss of consciousness or head trauma. The weakness was most prominent in the right upper extremity, accompanied by mild nausea and headache. She denied vision changes, speech difficulties, or seizures. Of note, due to her metastatic anal cancer, she was still undergoing her FOLFOX chemotherapy regimen, with the last cycle administered two weeks prior. She denied fevers, coughs, chest pain, or constitutional symptoms.

**Figure 1:**
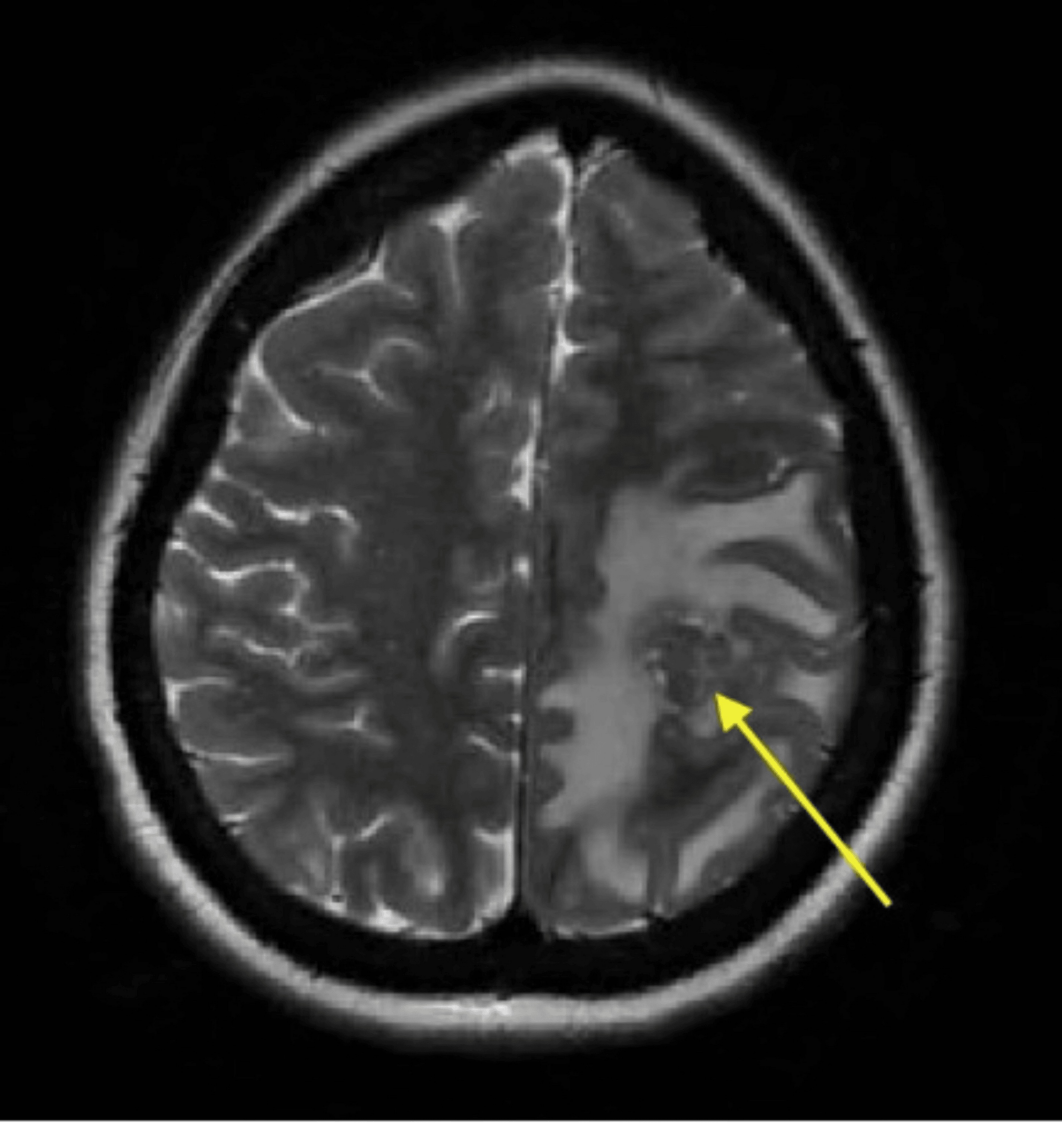
Axial T2-weighted MRI demonstrating a lesion in the left parietal lobe (yellow arrow) accompanied by surrounding vasogenic edema.

**Figure 2:**
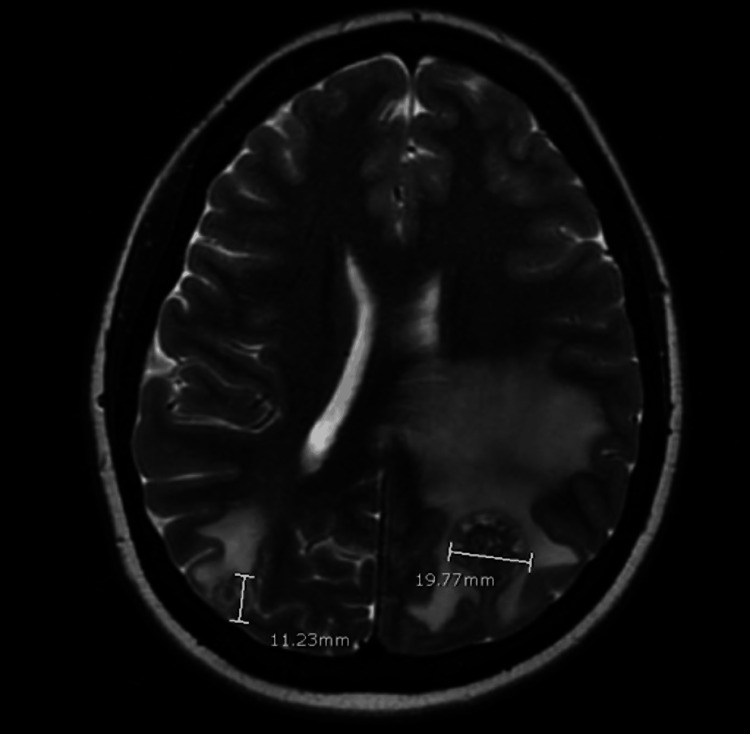
Axial T2-weighted MRI showing measurements of metastases: a right posterior parietal 1.1 x 1.2 x 1.0 cm lesion and a left parietal lobe lesion measuring 2.1 x 1.9 x 2.0 cm with left frontoparietal vasogenic edema surrounding the largest lesions, causing effacement of the left lateral ventricle and minimal focal rightward midline shift measuring 4.2 mm

**Figure 3:**
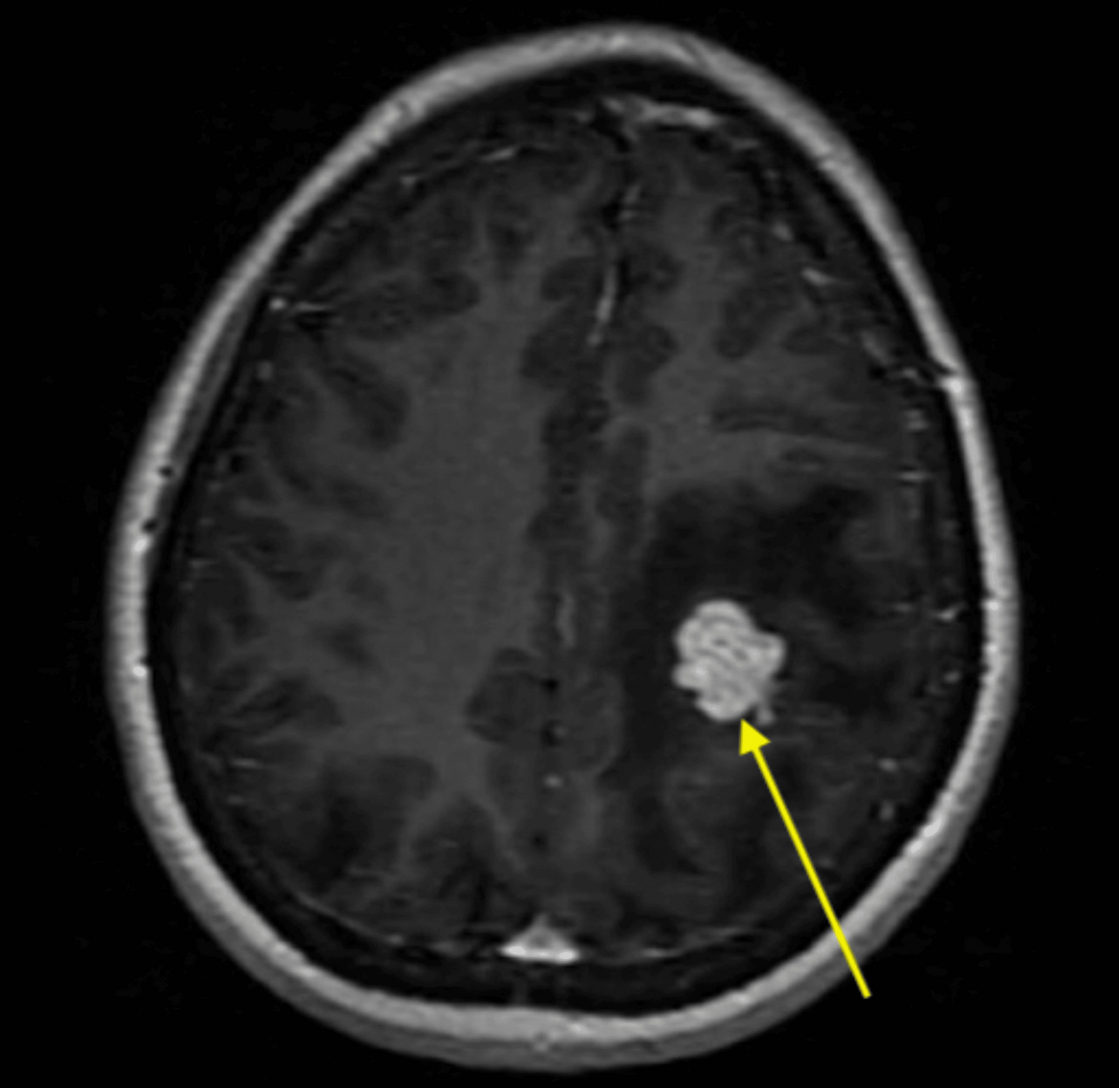
Axial T1-weighted post-contrast MRI showing an enhancing lesion in the left parietal lobe (yellow arrow) with surrounding vasogenic edema.

**Figure 4:**
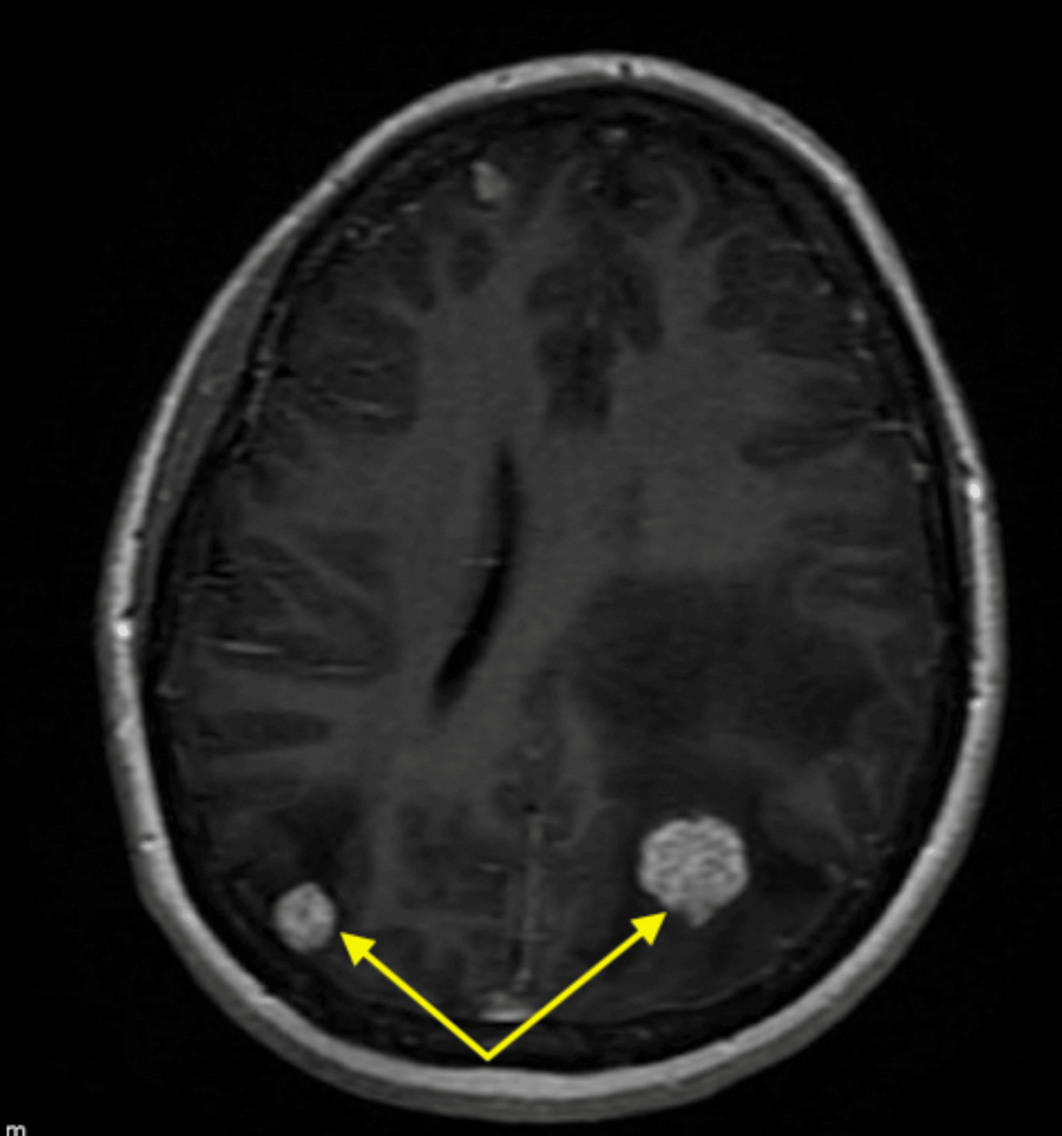
Axial T1-weighted post-contrast MRI showing enhancing lesions in the right posterior parietal and left parietal lobes (yellow arrows) with surrounding vasogenic edema.

Her initial workup included a non-contrast CT scan of the head, as an acute stroke was suspected; however, the CT was negative for intracranial hemorrhage, and initial testing did not reveal an acute ischemic process. The non-contrast head CT demonstrated left frontal and right posterior parietal vasogenic edema, raising concern for an underlying lesion. A subsequent contrast-enhanced CT and CT angiogram of the head showed bilateral metastatic lesions with surrounding vasogenic edema and mild rightward midline shift of approximately 4 mm, along with mild paranasal sinus mucoperiosteal thickening. An MRI was obtained to better assess the lesions, which showed multifocal bilateral enhancing lesions and left posterior parietal/occipital vasogenic edema. There was a mild focal right midline shift. Findings were consistent with metastatic disease. Multiple enhancing lesions throughout the brain parenchyma were seen: a left parietal lobe lesion measuring 2.1 x 1.9 x 2.0 cm, a 2.1 x 1.9 x 2.0 cm occipital lesion, a 0.9 x 1.1 x 1.1 cm left high convexity posterior frontal lesion, a right posterior parietal 1.1 x 1.2 x 1.0 cm lesion, and bilateral scattered subcentimeter enhancing lesions. There was left frontoparietal vasogenic edema surrounding the largest lesions and causing effacement of the left lateral ventricle and minimal focal rightward midline shift measuring 4.2 mm. A tiny enhancing right cerebellar lesion was also noted (Figures 1-4). 

Neurology was consulted, and the patient was started on intravenous dexamethasone 4 mg every six hours in the setting of vasogenic edema and levetiracetam 500 mg every 12 hours for seizure prophylaxis. With these treatments, her stroke-like symptoms fully resolved. Oncology and radiation oncology were consulted for the patient. Given metastatic lesions seen on imaging, the patient was planned for whole-brain radiotherapy, with plans to receive 39.6 Gy/22 fractions at 1.8 Gy per fraction. She was discharged on an oral dexamethasone taper (4 mg once daily for three days, then 2 mg once daily for four days, then 1 mg once daily for four days) with outpatient follow-up for ongoing radiation and oncology care.

## Discussion

Metastatic spread of anal SCC to the brain is uncommon, with a limited number of reported cases [4-8]. Our case is exceedingly rare considering our patient's negative HIV status and young age (only 27 years of age on admission, originally diagnosed at 26 years of age). To the best of our knowledge, no articles to date document brain metastases likely originating from anal SCC in a patient as young as 27. Tissue from the brain lesions was not obtained; therefore, confirmation of HPV status in the intracranial disease is lacking. This represents a limitation of the report. However, the presence of multiple lesions, known malignancy, and rapid resolution of symptoms with intravenous dexamethasone treatment also favored metastatic disease with vasogenic edema. Our patient’s known risk factors included HPV 16 positivity on Pap exam and sexual practices that included unprotected vaginal, oral, and anal sexual intercourse. Importantly, HPV 16 is the cause of most HPV-related anal cancer [9]. This underscores the importance of preventative care through early vaccinations targeted against HPV 16 and HPV 18, and other high-risk strains [4-5,7,9]. 

In this patient, the presentation of unilateral weakness initially raised suspicion for an acute stroke. However, MRI revealed multiple enhancing lesions consistent with metastases, likely hematogenous in origin. Despite the rarity of anal cancers metastasizing to the brain [4-8], it is imperative to obtain imaging in the context of neurological findings in patients with metastatic cancer [2, 4-8]. The imaging modality of choice is MRI [2]. Vasogenic edema surrounding metastatic lesions can cause focal neurological deficits and mimic cerebrovascular events [2]. Corticosteroids such as dexamethasone remain the cornerstone of management to reduce intracranial pressure and improve symptoms as they stabilize the blood-brain barrier, thereby lowering the intracranial pressure and improving neurological symptoms [2,10]. Whole-brain radiotherapy remains a standard treatment for multifocal lesions, while stereotactic radiosurgery may be considered for limited disease [2]. In younger patients, aggressive multimodal therapy, including radiation and systemic therapy, can offer symptomatic relief and potential survival benefit; however, brain metastases indicate an overall poor prognosis [1-8].

## Conclusions

Metastatic spread of anal SCC to the brain is rare, especially in a young patient. Our case is the first, to our knowledge, that documents an HIV-negative, HPV 16-positive female in her 20s with anal SCC with metastases to the brain. This further bolsters the importance of preventative care with HPV vaccinations to help protect against HPV 16, 18, and other high-risk strains. Additionally, this case highlights the necessity of brain imaging to rule out intracranial metastases in patients with malignancies presenting with new neurological deficits, even in cancers with low rates of brain metastasis, such as anal cancer.
